# Integrated Study of Transcriptome-wide m^6^A Methylome Reveals Novel Insights Into the Character and Function of m^6^A Methylation During Yak Adipocyte Differentiation

**DOI:** 10.3389/fcell.2021.689067

**Published:** 2021-12-03

**Authors:** Yongfeng Zhang, Chunnian Liang, Xiaoyun Wu, Jie Pei, Xian Guo, Min Chu, Xuezhi Ding, Pengjia Bao, Qudratullah Kalwar, Ping Yan

**Affiliations:** ^1^ Key Laboratory of Yak Breeding Engineering Gansu Province, Lanzhou Institute of Husbandry and Pharmaceutical Sciences, Chinese Academy of Agricultural Sciences, Lanzhou, China; ^2^ State Key Laboratory of Grassland Agro-Ecosystems, College of Pastoral Agriculture Science and Technology, Lanzhou University, Lanzhou, China

**Keywords:** yak, adipocyte, N6-methyladenosine, MeRIP-seq, regulatory mechanism

## Abstract

Yak (*Bos grunniens*) is considered an iconic symbol of Tibet and high altitude, but they suffer from malnutrition during the cold season that challenges the metabolism of energy. Adipocytes perform a crucial role in maintaining the energy balance, and adipocyte differentiation is a complex process involving multiple changes in the expression of genes. *N* ^6^-methyladenosine (m^6^A) plays a dynamic role in post-transcription gene expression regulation as the most widespread mRNA modification of the higher eukaryotes. However, currently there is no research existing on the m^6^A transcriptome-wide map of bovine animals and their potential biological functions in adipocyte differentiation. Therefore, we performed methylated RNA immunoprecipitation sequencing (MeRIP-seq) and RNA sequencing (RNA-seq) to determine the distinctions in m^6^A methylation and gene expression during yak adipocyte differentiation. In yak adipocyte and preadipocyte the content of m^6^A and m^6^A-associated enzymes was substantially different. In the two groups, a total of 14,710 m^6^A peaks and 13,388 m^6^A peaks were identified. For the most part, m^6^A peaks were enriched in stop codons, 3′-untranslated regions, and coding regions with consensus motifs of GGACU. The functional enrichment exploration displayed that differentially methylated genes participated in some of the pathways associated with adipogenic metabolism, and several candidate genes (*KLF9*, *FOXO1*, *ZNF395*, and *UHRF1*) were involved in these pathways. In addition to that, there was a positive association between m^6^A abundance and levels of gene expression, which displayed that m^6^A may play a vital role in modulating gene expression during yak adipocyte differentiation. Further, in the adipocyte group, several methylation gene protein expression levels were significantly higher than in preadipocytes. In short, it can be concluded that the current study provides a comprehensive explanation of the m^6^A features in the yak transcriptome, offering in-depth insights into m^6^A topology and associated molecular mechanisms underlying bovine adipocyte differentiation, which might be helpful for further understanding its mechanisms.

## Introduction

*N* ^6^-methyladenosine (m^6^A) was first discovered in the 1970s as the most prevalent internal modification of polyadenylated mRNAs and long noncoding RNAs (lncRNAs) in higher eukaryotes (Desrosiers et al., 1974; Perry and Kelley, 1974; Adams and Cory, 1975; Furuichi et al., 1975; Lavi and Shatkin, 1975; Wei and Moss, 1975). The modification of m^6^A methylation is mounted by a series of m^6^A methyltransferases labeled as writers: methyltransferases such as 3 and 14 (METTL3 and METTL14), Wilms Tumor 1-associated protein (WTAP), VIRMA, vir-Like m^6^A methyltransferase associated (KIAA1429), RNA binding motif protein 15 (RBM15), and zinc finger CCCH domain 13 (ZC3H13) (Bokar et al., 1997; Agarwala et al., 2012; Liu et al., 2014; Ping et al., 2014; Schwartz et al., 2014; Patil et al., 2016; Knuckles et al., 2018; Wen et al., 2018). Besides this, m^6^A demethylases eliminate methylation from RNAs to enable a delicately dynamic equilibrium modification and are named erasers: fat mass and obesity-associated protein (FTO) and α-ketoglutarate-dependent dioxygenase alkB homolog 5 (ALKBH5) (Jia et al., 2011; Zheng et al., 2013). Further, specific proteins, including the YTH domain family (YTHDF1-3) and IGF2BPs (IGF2BP1-3) (Dominissini et al., 2012; Luo and Tong, 2014; Wang et al., 2014; Wang et al., 2015), were identified as a category of proteins called readers that recognize the information of RNA methylation modifications and engage in downstream mRNA translation, degradation, microRNA binding, and RNA-protein interactions (Liu and Pan, 2016; Roundtree I. A. et al., 2017; Nachtergaele and He, 2017; Zhao et al., 2017). Notably, two independent studies established an m^6^A RNA immunoprecipitation accompanied with high-throughput sequencing (MeRIP-seq) and subsequently identified the first *N* ^6^-methyladenosine modification map to methylomes with a resolution of 100-nucleotides (Dominissini et al., 2012; Meyer et al., 2012). Meanwhile, MeRIP-seq has been used to identify the m^6^A profile in humans and mice. These results reveal that m^6^A is predominantly located close to stop codons, 3′-untranslated regions (3′-UTRs), and also in long internal exons and transcription start sites, suggesting that m^6^A plays a crucial role in the post-transcriptional regulation of gene expression. These innovative studies reflect that the construction of transcriptome-wide m^6^A methylome profiles is of great importance to further investigate the characteristics and functions of such modification.

Currently, m^6^A modifications are reported in several areas of RNA metabolism, such as RNA localization, transport, splicing, stability, and translation (Liu and Pan, 2016; Roundtree I. A. et al., 2017; Nachtergaele and He, 2017; Zhao et al., 2017). Previous studies describe that m^6^A modification of mRNA plays an important biological function in controlling cellular metabolic processes, and it is reportedly involved in determining mammalian embryonic stem cell fate (Batista et al., 2014), regulating the initiation and differentiation of meiosis in murine spermatogonial stem cells (Xu et al., 2017), and maintaining the myogenic potential of proliferating skeletal muscle progenitors (Kudou et al., 2017). In particular, FTO facilitates the differentiation of mouse preadipocytes by regulating alternative splicing of pre-mRNAs for genes associated with adipogenesis (Zhao et al., 2014). Zhong et al. report that knockdown of METTL3 or YTHDF2 *in vitro* enhanced the stability and expression of peroxisome proliferator-activator receptor alpha (PPARα) mRNA, leading to decreased lipid accumulation in a hepatocellular carcinoma cell line (HepG2) (Zhong et al., 2018). Besides this, a recent study reveals that RNA m^6^A modification has a potential function in the deposition of porcine adipose tissue (Tao et al., 2017), and the modification of m^6^A on the mRNA of mitochondrial carrier homology 2 (MTCH2) promotes the differentiation of pig intermuscular preadipocytes (Jiang et al., 2019). Thus, we assume that m^6^A modification may also refer to bovine adipocyte differentiation according to the notable functions of m^6^A modification described above. However, our knowledge about the relationship between m^6^A modification and bovine adipocyte differentiation is still scarce.

The yak is the major bovine livestock breed on the Qinghai-Tibet Plateau and is the only large ruminant domestic species that enables daily necessities, such as meat, milk, wool, skins, fuel, and economic benefits, for local herders (Long et al., 1999; Dong et al., 2006). On the Qinghai-Tibet Plateau, domestic yaks mainly grow on natural pastures under typical grazing conditions (Long et al., 2008). Owing to seasonal variations in forage, yaks must constantly undergo insufficient feeding during the harsh winter season (October–May), which leads to the large seasonal weight changes and a circular rhythm of “live in summer, weighty in autumn, thin in winter, and dead in spring” (Shikui et al., 2003). Consequently, the subcutaneous adipose layer of yak accumulates rapidly in summer and early autumn to provide essential energy requirements and withstand severe cold through selective fat catabolism during the cold season (Ding et al., 2012). The distinctive metabolic pattern makes the yak a fascinating model for studying adipose metabolism in plateau domestic animals. Adipocytes are a major component of adipose tissue and are considered to be the cornerstone of metabolic homeostasis regulation throughout the body (Ali et al., 2013). Therefore, it is necessary to assay m^6^A sites at the transcriptome-wide level to identify the potential biological functions of RNA m^6^A modification during yak adipocyte differentiation.

In the present study, we initially isolated preadipocytes from yak adipose tissue and differentiated them into mature adipocytes successfully. We obtained the first transcriptome-wide m^6^A methylome profile in yak by MeRIP-seq and elucidated the features of m^6^A modification during yak adipocyte differentiation. We found that the different m^6^A RNA modifications between yak preadipocytes and mature adipocytes have potential regulatory roles in gene expression and pathways related to adipose energy metabolism. This study explores the role of m^6^A modification in bovine adipose metabolism and complements m^6^A studies in plateau domestic livestock, which may be a breakthrough point for exploring energy metabolism in yaks.

## Materials and Methods

### Ethics Statement

Animal treatment during research was carried out in complete accordance with the protocols and guidelines for animal ethics of the People’s Republic of China, and all operations were approved by the Animal Administration and Ethics Committee of Lanzhou Institute of Husbandry and Pharmaceutical Sciences, Chinese Academy of Agricultural Sciences (Permit No. SYXK-2014–0002).

### Preadipocyte Isolation

The Datong Yak Breeding Center (Datong County, Qinghai, China) provided three healthy 3-day-old Datong yaks. The night before slaughter, yaks were not fed. On the next morning, the yaks were humanely sacrificed by the way of electrical stunned (90 V, 10 s, and 50 Hz) at a commercial slaughter facility and exsanguinated as necessary to ameliorate the suffering, according to standard approved industry protocols. The subcutaneous adipose tissue was harvested according to the protocols and guidelines for animal ethics of the People’s Republic of China. Primary yak preadipocytes were cultured from subcutaneous adipose tissue according to our previous study (Zhang Y. et al., 2018; Zhang et al., 2020). Briefly, the subcutaneous fat tissue was flushed with penicillin (200 U/mL) and streptomycin (200 U/mL) added to the phosphate saline buffer (HyClone, Thermo Fisher Scientific, Carlsbad, CA, United States). After that, they were finely minced into about 1 mm^3^ piece in an aseptic setting. The segments were digested by Type I collagenase in a continuously agitated water bath at 37°C for 60–90 min. With a 40-μm nylon mesh film, indigestible material was screened, and the filtrate was resuspended for 5 min at 1400 g. The sediment was subsequently incubated at room temperature for 10 min with the erythrocyte lysis buffer (0.154 M NH4Cl, 10 mM KHCO_3_, 0.1 mM EDTA). The cells were then filtered with 200-μm nylon mesh film and rinsed twice with a serum-free medium. After 5 min of centrifugation at 1400 g, preadipocytes were harvested and solubilized in the growth media, including DMEM-F12 (Hyclone, UT, United States) supplemented with 10% fetal bovine serum (FBS, Gibco, MA, United States).

### Adipogenic Differentiation and Staining of Oil Red O

The adipogenic differentiation was performed according to our previous study (Zhang Y. et al., 2018; Zhang et al., 2020). Preadipocyte was induced for 2 days by adipogenic compounds composed of 3-isobutyl-methylxanthine (MIX) (Sigma, MO, United States), dexamethasone (Sigma, MO, United States), rosiglitazone (Sigma, MO, United States), and insulin (Sigma, MO, United States) after cell confluence approached 70% in growth media. The medium was replaced after 2 days with DMEM-F12 containing 10% FBS, penicillin (200 U/mL), streptomycin (200 U/mL), and 5 ng/ml of insulin and updated with cycles of 2 days until day 12. The cells were usually flushed twice with PBS and set for 1 h in 4% formalin. Cells were then reacted at room temperature for 30 min with a saturated solution of Oil Red O. Then, cells were rinsed three times with sterile water, and photographs were acquired from light microscopy.

### Quantitative Real-Time PCR

Total RNAs were extracted using TRIzol reagent (Invitrogen, CA, United States) from *in vitro* cultured yak preadipocytes and differentiated adipocytes (three biological replicates for each condition). Concentration and quality were further evaluated using denaturing gel electrophoresis and spectroscopy (Thermo, Waltham, MA, United States). Reverse transcription of mRNA was conducted using commercial kits (Takara, Japan) according to the manufacturer’s protocols. Real-time RT-PCR was accomplished in a CFX Link Real-Time PCR Detection System, and 10 μl volume of reaction consisting of 5 μl 2xSYBR Premix Ex Taq II, 0.4 μl primers (10 μM), and 0.8 μl cDNA. The reaction condition was as follows: denaturation for 30 s at 95°C followed by 35 additional cycles for 15 s at 94°C, annealing for the 30 s at 72°C. A melting procedure with a heating rate of 0.5°C/10 s was performed to create melting curves ranging from 95°C. The gene expression levels were estimated using the 2^−ΔΔCt^. Supplementary Table S1 lists the sequences used for the primers.

### Measuring the m^6^A Content

The overall content of mRNA m^6^A was measured by a methylation quantification kit of EpiQuik RNA (Epigentek, P-9005, NY, United States). In short, a standard curve was constructed at concentrations of 0.01–0.5 ng/μl by positive control. The equivalent RNA solution (1–8 μl) and negative control were applied to the strip wells. The plate was wrapped with parafilm, incubating for 1.5 h at 37°C. Then, the wells were washed three times and added to the 1:1000 diluted capture antibody at room temperature for 1 h. After washing thrice, the detection antibody (1:2000 dilution) and enhancer solution were applied to every well incubated at room temperature for 30 min. After five washes, detection solutions were placed on each well and incubated for 10 min at room temperature to protect from light. Finally, a stop solution was applied to each well and absorbance read with a microplate reader at 450 nm.

### MeRIP-Seq and mRNA Sequencing

According to the manufacturer’s protocol, the total RNA was extracted using Trizol reagent (Invitrogen, CA, United States). A Bioanalyzer 2100 and RNA 6000 Nano LabChip Kit (Agilent, CA, United States) with RIN number >7.0 were used to evaluate the total RNA quality and quantity. Nearly over 200 μg total RNA was performed to isolate Poly (A) mRNA through magnetic beads (Invitrogen) attached to poly-T oligo. After purifying, poly (A) mRNA fractions are broken into 100-nt-long oligonucleotides using a Magnesium RNA Fragmentation Module (NEB, cat.E6150, United States) under 86°C for 7 min. Then, the fragmentation of broken RNA was incubated in immunoprecipitation (IP) buffer (50 mM Tris-HCl, 750 mM NaCl, and 0.5% Igepal CA-630) supplied with BSA (0.5–1 μg/μl) for 2 h at 4°C with m^6^A-specific antibody (No. 202003, Synaptic Systems, Germany). Subsequently, the above mixture was incubated with protein-A beads (Thermo Fisher Scientific, MA, United States) and eluted with elution buffer (1 × IP buffer and 6.7 mM m^6^A). The eluted RNA was extracted by TRIzol reagent (Thermo Fisher Scientific) according to the manufacturer. Then, IP RNA and untreated input control fragment RNA were reverse-transcribed to create the cDNA by SuperScript™ II Reverse Transcriptase (Invitrogen, cat. 1896649, CA, United States), which was then used to synthesize U-labeled second-stranded DNAs with *E. coli* DNA polymerase I (NEB, cat.M0209, MA, United States), RNase H (NEB, cat.M0297, MA, United States), and dUTP Solution (Thermo Fisher, cat. R0133, MA, United States). Besides this, the A-base was added to the blunt ends of each strand and prepared for linkage to the indexed adapters. Each adapter holds a T-base overhang to link the adapter to the A-tailed fragmented DNA. Single- or dual-index adapters were linked to the fragments, and size selection was performed with AMPureXP beads. After the heat-labile UDG enzyme (NEB, cat. M0280, MA, United States) treatment of the U-labeled second-stranded DNA, the ligated products are amplified with PCR by the following conditions: initial denaturation at 95°C for 3 min, eight denaturation cycles at 98°C for 15 s, annealing at 60°C for 15 s, extension at 72°C for 30 s, and then final extension at 72°C for 5 min. The average insert size of the paired-end libraries was ∼100 ± 50 bp. Finally, the m^6^A-seq libraries were performed with Tru Standard mRNA Sample Prep Kit (Illumina) along with the published protocol (Huse et al., 2003). The 2 × 150 bp paired-end sequenced (PE150) on Illumina Novaseq™ 6000 (LC-Bio Technology CO., Ltd., Hangzhou, China) in accordance with the vendor’s recommended protocol.

### Sequencing Data Analysis

First of all, in-house perl scripts and Cutadapt (Martin, 2011) were performed to eliminate the reads containing contaminants of the adapter, bases of low quality, and indeterminate. Meanwhile, the quality of the sequence was validated using fastp. The reads were mapped to the *Bos*_*mutus* genome (Version: BosGru_v2.0) by HISAT2 (Kim et al., 2015) with default parameters. Using R package exomePeak (Meng et al., 2014) identify the m^6^A peaks from mapped reads of IP and input libraries with bed or bam format to configure for viewing on IGV software (http://www.igv.org/) or the UCSC genome browser. The parameters of the exomePeak R package are as follows: “PEAK_CUTOFF_PVALUE = 0.05, PEAK_CUTOFF_FDR = NA, FRAGMENT_LENGTH = 100.” The examination was performed using the Poisson distribution model, and a *p*-value < 0.05 was considered as a peak. *De novo* and defined motifs were identified by MEME (Bailey et al., 2009) and HOMER (Heinz et al., 2010), accompanied by perl scripts in the house seeking the motif concerning peak. Called peaks were annotated using ChIPseeker (Yu et al., 2015) by intersection with gene architecture. The difference peaks were identified using the exomePeak R package with parameters *p*-value < 0.05 and |log2 (fold change)| ≥ 1. StringTie (Pertea et al., 2015) calculated the expression level of all mRNAs from input libraries, which normalized with FPKM {FPKM = [total exon fragments/mapped reads (millions)]}. The differentially expressed mRNAs were collected by R package edgeR (Robinson et al., 2010) with the |log2 (fold change)| > 1 and *p*-value < 0.05. GO seq R package was performed on the Gene Ontology (GO, http://www.geneontology.org/) enrichment analysis for the differentially expressed genes (Young et al., 2010). The Kyoto Encyclopedia of Genes and Genomes (KEGG, http://www.genome.jp/kegg/) database is a major resource for learning high-level functions and utilities of biological systems. The statistical enrichment tests for genes of differential expression in the KEGG pathways were used in the KOBAS software (Xie et al., 2011).

### Western Blotting

Proteins were extracted from preadipocytes and adipocytes. After detecting the total protein concentration, the protein was denatured at 95°C for 5 min with a protein loading of 50 μg. Subsequently, SDS-PAGE electrophoresis was performed with 10% of the isolate gel and 4% of the concentrate gel, and electrophoresis at 40 V for 25 min in the concentrate gel and 100 V for 80 min in the isolate gel. Then, the protein was transferred to the PVDF membrane and immersed in the closure solution at 37°C for 1.5 h. Then, it was incubated in monoclonal rabbit anti-ENTPD1, anti-USP2, and anti-PGAM2 (1:1000; Abcam, Cambridge, United Kingdom) and monoclonal mouse anti-β-actin (1:5,000; Beyotime, Shanghai, China). Finally, the membranes were incubated for 1.5 h at 37°C by adding an HRP-labeled goat secondary antibody and images captured using a Chemi Doc System (Bio-Rad, Hercules, CA). Grayscale values of proteins were evaluated by ImageJ (https://imagej.nih.gov/ij/).

### Statistical Analysis

The SPSS 22 software package was used to evaluate statistics. A one-way test of variance assessed the significance of the differences between all of the groups. Statistically significant was the degree of probability * *p* < 0.05;** *p* < 0.01. Values are shown as mean ± SEM.

## Results

### The Yak Preadipocyte Induced Differentiation and Global m^6^A Quantification

**FIGURE 1:**
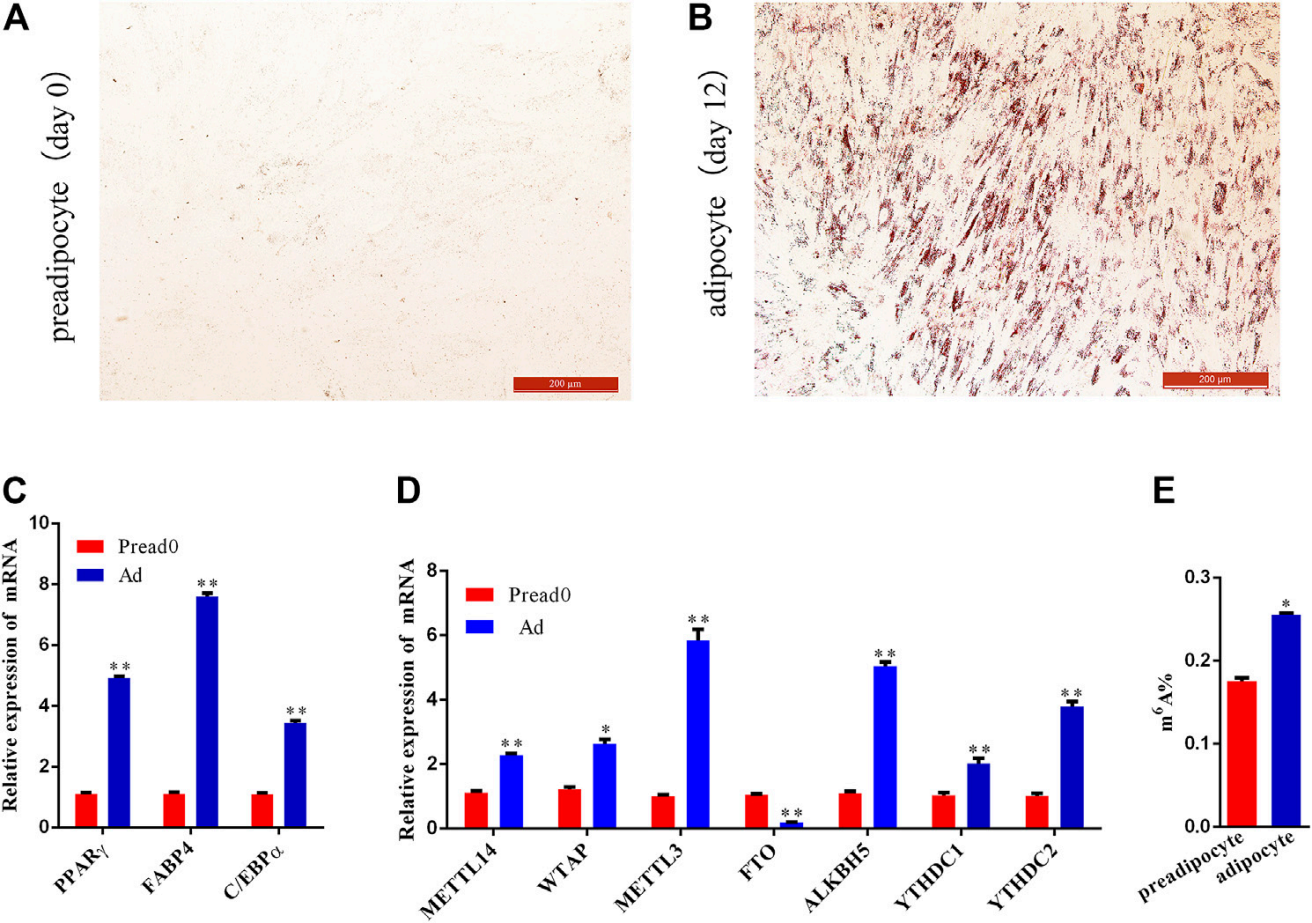
Preadipocyte-induced differentiation and detection of m^6^A level between preadipocyte and adipocyte. **(A,B)** Preadipocytes and adipocytes dyed with Oil red O. **(C)** Relative expression of *PPARγ*, *C/EBPα*, and *FABP4* between preadipocyte (day 0) and adipocyte (day 12). **(D)** The mRNA expression level of methyltransferases (*METTL14*, *WTAP*, and *METTL3*), demethylases (*FTO* and *ALKBH5*) and m^6^A-binding proteins (*YTHDC1*and *YTHDC2*). **(E)** m^6^A abundance between the preadipocytes and adipocytes. (SEM, * *p* < 0.05;** *p* < 0.01).

The results of Oil Red O show that the visibility of lipid droplets in adipocytes increased significantly at day 12 compared to day 0 after induction with adipogenic agents (Figures 1A,B, Supplementary Figure S1). Meanwhile, the expression of adipocyte differentiation-specific marker genes (*PPARγ*, *C/EBPα*, and *FABP4*) was significantly elevated on day 12 (adipocyte) compared with day 0 (preadipocyte) (Figure 1C), suggesting preadipocyte full differentiation into adipocyte. Subsequently, to overview the m^6^A methylation during yak adipocyte differentiation, the expression of RNA methylation-related genes was contrasted by quantitative real-time PCR (qRT-PCR) detected, including *METTL3*, *WTAP*, *METTL14*, *FTO*, *ALKBH5*, and *YTHDC1/2*. Comparing the group of preadipocytes (Pread0) and adipocytes (Ad), the findings show that the expression level of methyltransferases (*METTL14*, *WTAP*, and *METTL3*) and *ALKBH5* were dramatically upregulated, whereas *FTO* was substantially downregulated, and m^6^A-binding proteins (*YTHDC1* and *YTHDC2*) were drastically upregulated (Figure 1D). Furthermore, the content of m^6^A in the group of adipocytes was significantly higher compared with the preadipocyte group (Figure 1E). Thereby, we hypothesized that, during yak adipocyte differentiation, the difference of m^6^A methylation may exist, which was furtherly discovered using MeRIP-seq.

### Transcriptome-wide m^6^A-Seq Reveals Global m^6^A Modification Patterns During Yak Adipocyte Differentiation

**FIGURE 2:**
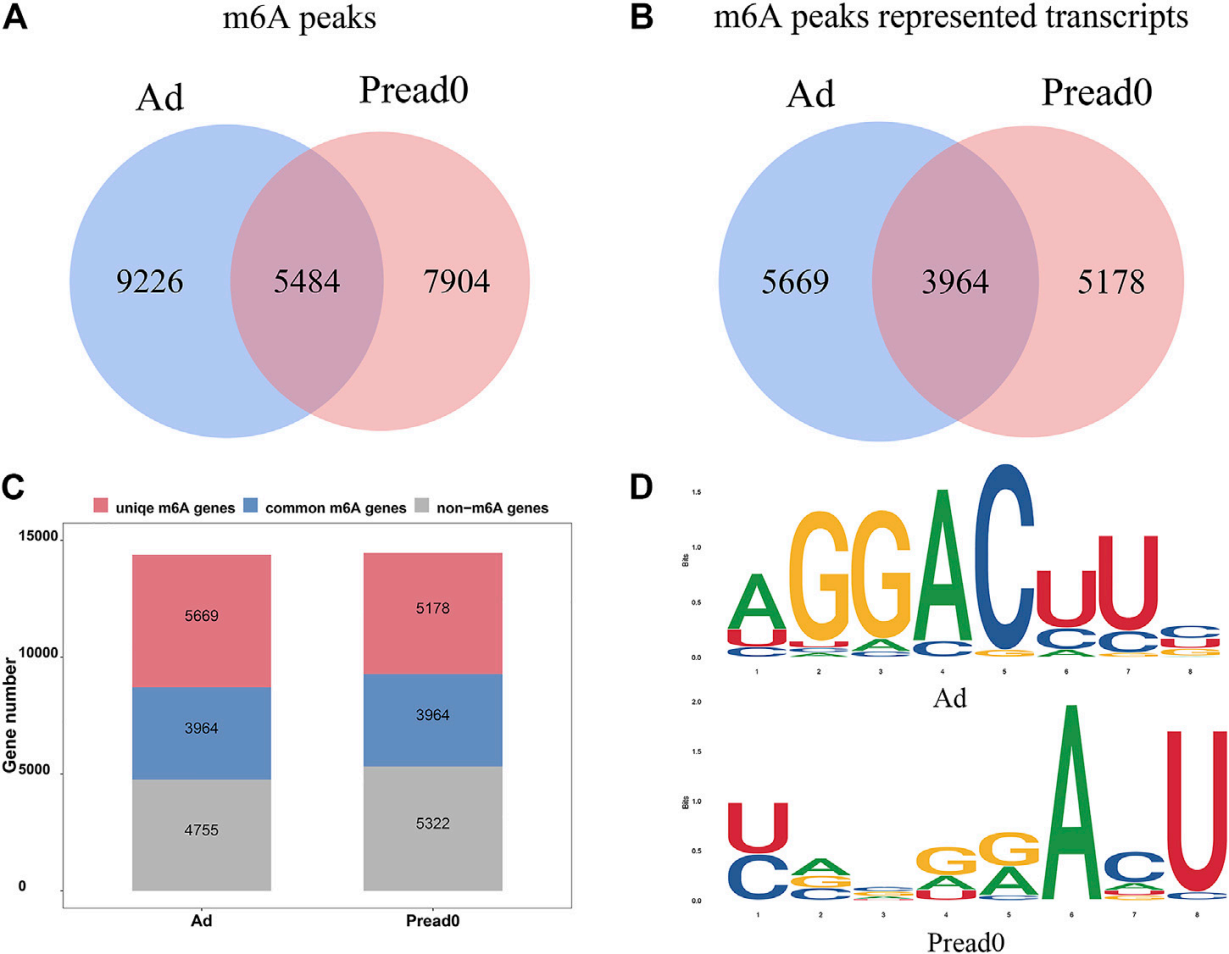
m^6^A-seq transcriptome-wide and m^6^A peak analysis. **(A)** Number of common and specific m^6^A peaks in groups of preadipocyte and adipocyte. **(B)** Venn diagram showing the m^6^A peaks for transcripts of two group. **(C)** Profile of m^6^A-modified genes discovered in m^6^A-seq. **(D)** The motifs enriched from m^6^A peaks identified from the groups of adipocyte and preadipocyte.

The yak adipocyte and preadipocyte of three biological replicates were used for transcriptome-wide m^6^A-sequencing (m^6^A-seq) and RNA-sequencing (RNA-seq) assays. In total, 12 libraries were sequenced, comprising three replicates of preadipocyte and adipocyte for input and MeRIP samples (Supplementary Table S2). With each MeRIP library, an average of 9.22 Giga base-pair (Gb) of high-quality data was produced, and 9.49 Gb per input library (RNA-seq data set). Then, we eliminated reads containing adapter pollutants, low quality, and indeterminate bases, an average of 7.17 and 7.11 Gb obtained from per MeRIP and input libraries, respectively. The valid data were mapped to the *Bos*_*mutus* genome (Version: BosGru_v2.0) using HISAT2. The proportions of mapped reads ranged from 87.96 to 96.57%, correspondingly (Supplementary Table S2). The RNA species of transcripts included mRNA (19,916), misc_RNA (386), ncRNA (262), pseudogene (916), and tRNA(179) (Supplementary Figure S2). In the yak Ad group, R package exomePeak found a total of 14,710 m^6^A peaks, containing transcripts of 9633 genes. Likewise, 13,388 m^6^A peaks were found in the Pread0 group corresponding to transcripts of 9142 genes (Figures 2A,B). In addition, 5848 peaks were consistently observed in the two groups, and 3964 genes within the groups were modified by m^6^A. Compared with the Pread0 group, the Ad group had 9226 new peaks occurring with the absence of 7904 peaks, reflecting the significant difference between Pread0 and Ad groups in global m^6^A modification trends (Figures 2A–C). m^6^A methylomes were ulteriorly mapped by HOMER software to define whether RRACH motifs (R represents purine; A is m^6^A; and H is U, A, or C) were ubiquitous in our detected m^6^A. The results of the enrichment analysis in both groups show that the consensus motifs of m^6^A RRACH were GGACU (Figure 2D) accorded with previous studies, which strengthens the credibility of the m^6^A peaks and confirms the presence of a prevailing methylated modification mechanism.

### Analysis of m^6^A Modification Distribution in Yak Transcriptome

**FIGURE 3:**
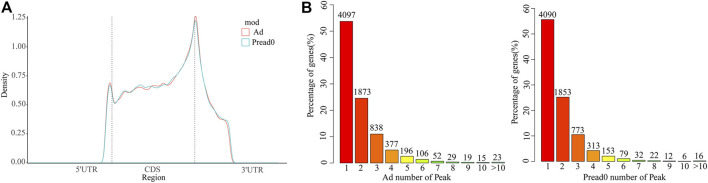
m^6^A methylome distributed on yak transcripts. **(A)** m^6^A peaks enriched with transcripts. Transcript is separated into three parts comprising 5′ untranslated region, coding sequence and 3′ untranslated region. **(B)** Proportion of genes possessing m^6^A peaks with different numbers.

We analyzed metagene models of m^6^A peaks in the global transcriptome to identify the differential distribution of m^6^A in transcripts. Our findings indicated that m^6^A peaks were predominantly enriched in the coding sequence (CDS) near the start and stop codons and approach the beginning of the 3′ untranslated region (3′UTRs) in Ad and Pread0 (Figure 3A), which contrast to the pattern found in mice and chickens (Luo et al., 2019; Cheng et al., 2021). Subsequently, to systematically calculate the enrichment, we investigated nonoverlapping transcript segments per m^6^A peak with 5′UTR, CDS, and 3′UTR (Supplementary Figure S3A), in which most of them were abundant in CDS. Interestingly, m^6^A peak relative increased at 5′UTR and CDS region in Ad compared with Pread 0 and decreased in 3′UTR region. Afterward, we explored the distribution of m^6^A modified peaks with each gene, finding that almost 60% of methylated genes hold only one m^6^A peak, and most genes contain one to three m^6^A peaks (Figure 2B). Furthermore, we investigated the relationship between m^6^A peak number and gene length. The results show a global trend that the longer gene length has more m^6^A peaks (Supplementary Figure S3B).

### Analysis of the GO and KEGG Pathways of Differentially Methylated Genes

**FIGURE 4:**
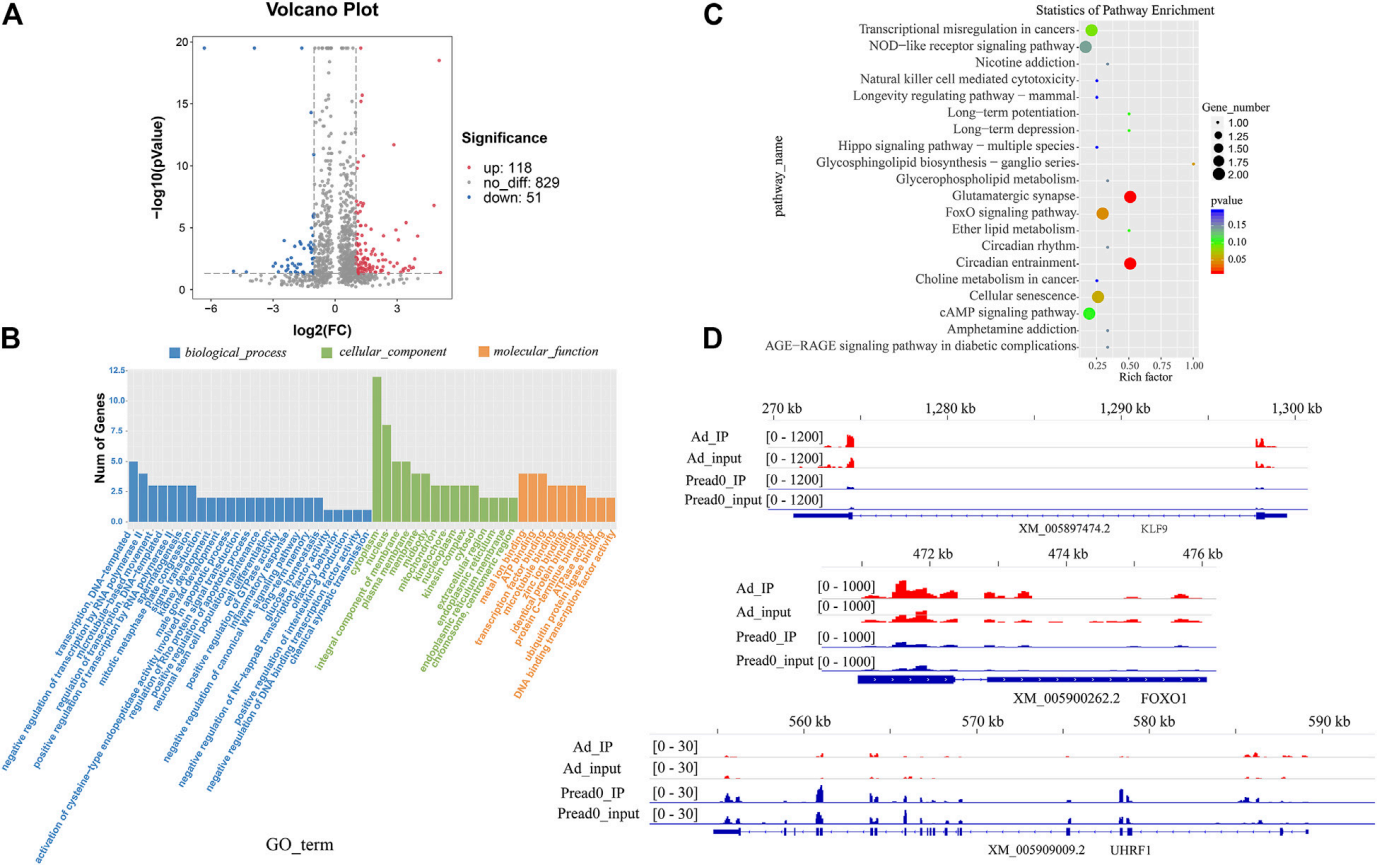
The alteration of global m^6^A modification in adipocyte compared with preadipocyte. **(A)** Volcano plots showing different m^6^A peaks (fold change ≥2 and *p* < 0.05) between preadipocyte and adipocyte. **(B)** GO terms of genes with differential m^6^A peaks between preadipocyte and adipocyte. **(C)** The top 20 markedly enriched pathways for the genes of differential peaks between preadipocyte and adipocyte. **(D)** The abundance of m^6^A on *KLF9*, *FOXO1*, and *UHRF1*mRNA transcripts observed by m^6^A-seq in preadipocyte and adipocyte. Blue boxes represent exons; blue lines represent introns.

The comparison was performed for the abundance of m^6^A peaks between preadipocytes and adipocytes. These findings exposed that 118 markedly hypermethylated m^6^A peaks and 51 substantially hypomethylated peaks were obtained (|log2 (fold change)| > 1, *p*< 0.05) (Figure 4A). The residual peaks of the m^6^A were viewed as unaltered peaks. Moreover, differentially methylated m^6^A peaks represented genes investigated by GO and KEGG pathway analysis, revealing the biological significance of m^6^A methylation during yak adipocyte differentiation. GO analysis revealed that differentially methylated genes were mainly implicated with DNA-templated and regulation of transcription by RNA polymerase II (ontology: biological process), cytoplasm, nucleus and integral component of membrane (ontology: cellular component), and transcription factor and microtubule binding (ontology: molecular function) (Figure 4B, Supplementary File S1). Meanwhile, the top 20 biological enrichment of KEGG pathways indicated that the genes differently methylated were substantially related to the adipogenic metabolism regulation pathways, NOD-like receptor signaling pathway, FoxO signaling pathway, Ether lipid metabolism, cAMP signaling pathway, and Hippo signaling pathway (Figure 4C; Supplementary File S2). These results reveal that several genes related to lipid metabolism were modified by m^6^A methylation during yak adipocyte differentiation. Furthermore, the genes (*KLF9*, *FOXO1*, and *UHRF1*) differentially methylated sites were analyzed by Integrative Genomics Viewer (IGV) software (Figure 4D), located in 5′UTRs, exons, and 3′UTRs. In the 5′UTR region of *KLF9*, the m^6^A site was hypermethylated in the adipocyte group compared with the control group, and its mRNA expression was upregulated. In the 3′UTR region of *FOXO1*, the m^6^A site was hypomethylated in the adipocyte group compared with the control group, and its mRNA expression was upregulated. In the exon region of *UHRF1*, the m^6^A site was hypermethylated in the adipocyte group compared with the control group, and its mRNA expression was downregulated (Supplementary File S3). The different m^6^A methylation levels of these genes may affect their expression.

### RNA-Seq Identification of Genes Differentially Expressed in Both Groups

**FIGURE 5:**
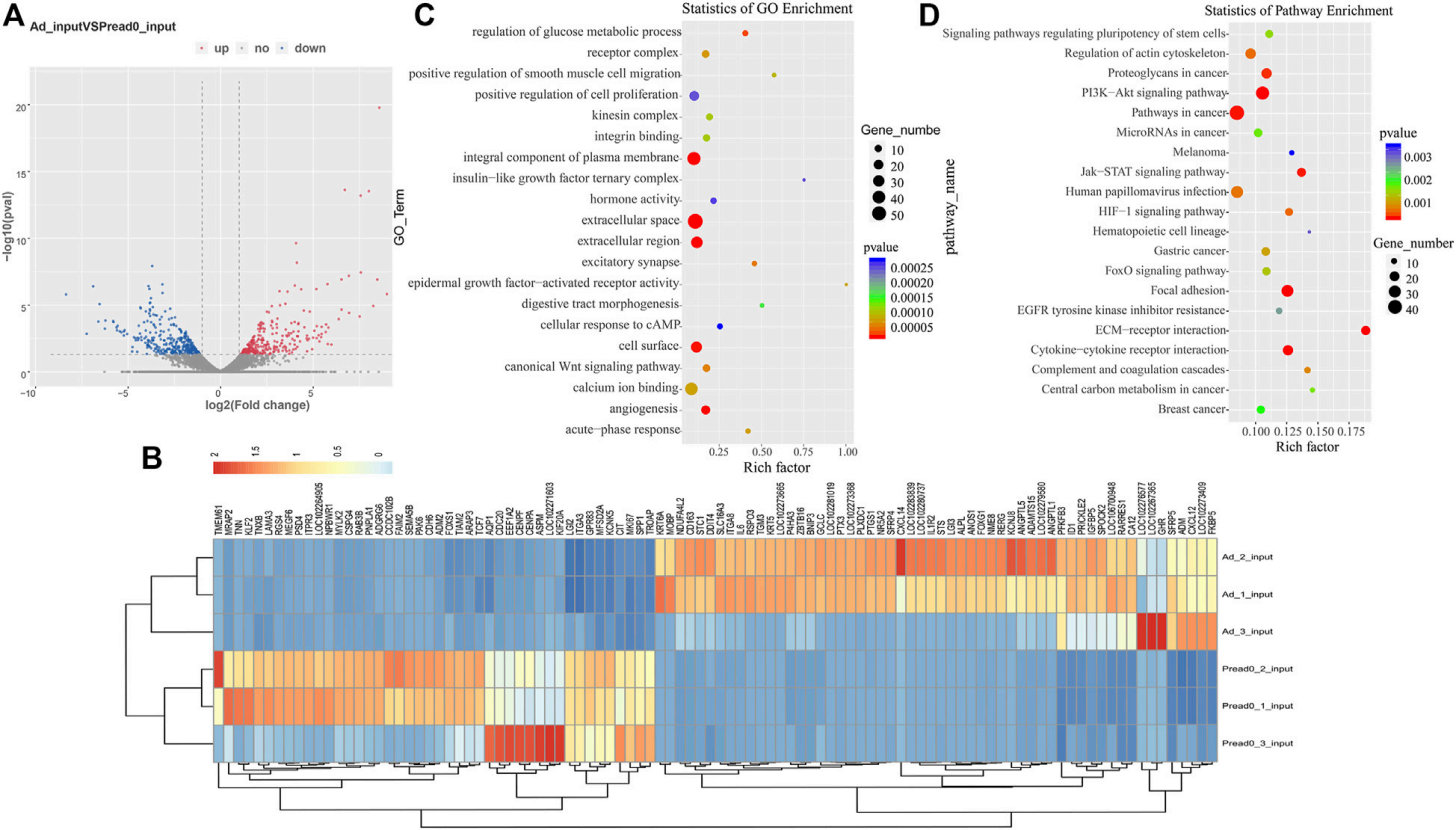
RNA-seq analysis of genes differentially expressed between preadipocyte and adipocyte. **(A)** Volcano plots illustrating the differentially expressed genes. **(B)** Clustered heat map for comparing preadipocyte and adipocyte with the top 100 most differentially expressed genes. **(C)** The top 20 significantly GO enrichment terms for significantly differentially expressed genes. **(D)** The top 20 enriched pathways of dramatically differentially expressed genes.

An analysis of the RNA-seq data set (m^6^A-seq input library) displayed that the trends of global mRNA expression between preadipocyte and adipocyte were considerably different. There were 648 significantly different mRNAs, including 300 upregulated and 348 downregulated (|log2 (fold change)| > 1, *p* < 0.05) as shown in Figure 5A. Then, we conducted a clustered heat map to further explore the potential roles of the genes (Figure 5B; Supplementary File S4). Furthermore, GO ontology and KEGG pathway were performed to analyze the differentially expressed genes. As Figure 5C; Supplementary File S5 display, the top 20 most notable functional annotations include regulation of glucose metabolic process, canonical Wnt signaling pathway, positive regulation of cell proliferation, and insulin-like growth factor ternary complex, which influence adipocyte differentiation. Meanwhile, the pathway exploration revealed that signaling pathways regulating pluripotency of stem cells, ECM-receptor interaction, PI3K-Akt signaling pathway, and FoxO signaling pathway were significantly enriched (Figure 5D; Supplementary File S6), revealing that differentially expressed genes potentially participated in adipogenic metabolism.

### Conjoint Analysis of RIP-Seq and RNA-Seq Data With Both Groups

**FIGURE 6:**
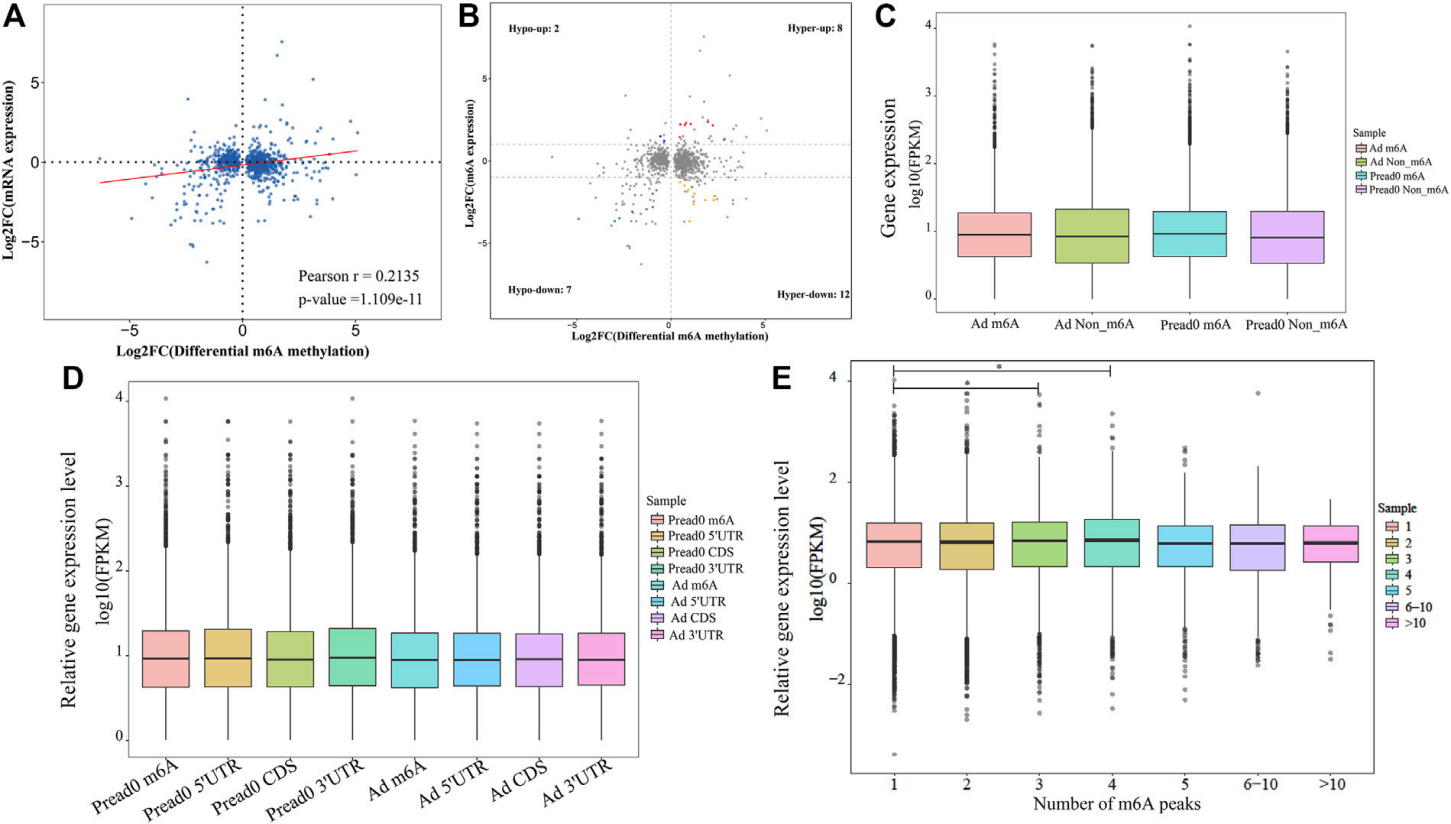
Conjoint analysis of differentially methylated genes and differentially expressed genes. **(A)** Dot plot of Log_2_ fold change (FC) (mRNA expression) versus Log_2_ FC (differential m^6^A methylation) revealing a positive association between total m^6^A methylation and level of mRNA expression (Pearson r = 0.2135; *p*-value = 1.109e-11). **(B)** Four-quadrant plot showing differentially expressed genes with differentially methylated m^6^A peaks. **(C)** The holistic expression levels of the transcripts with m^6^A-methylated and non-m^6^A-methylated in different groups. **(D)** For all m^6^A peaks, the position of m^6^A peaks for RNA transcripts and gene relative expression level. **(E)** Relative mRNA transcript expression level with different number of m^6^A peaks. *p < 0.05 compared with the m^6^A peak = 1.

We found an interesting relationship of differentially methylated m^6^A peaks and gene expression patterns in preadipocytes and adipocytes through cross-analysis of the m^6^A-seq and RNA-seq results, in which a positive correlation existed in differentially methylated m^6^A peaks and gene expression levels (Figure 6A). Otherwise, all genes were segregated into mainly four types: eight hypermethylated and upregulated genes termed “hyper-up”; seven hypomethylated and downregulated genes termed “hypo-down”; 12 hypermethylated while downregulated genes termed “hyper-down”; and two hypomethylated while upregulated genes termed “hypo-up” (Figure 6B). There were slightly more hyper-up and hypo-down than hyper-down and hypo-up. Table 1 lists the expression of genes that were significantly differently (|log2 (fold change)| > 1, *p*<.05), comprising significantly differently methylated peaks. Then, both groups were evaluated for the overall expression levels of the m^6^A-methylated and non-m^6^A-methylated transcripts (Figure 6C); the expression of methylated transcripts was higher than that of nonmethylated transcripts. These suggest that, in yak adipocyte differentiation, m^6^A modifications appear to have a positive association with mRNA expression. Furthermore, we were wondering if the position of m^6^A peaks on RNA transcripts or the number of m^6^A peaks per transcript is correlated with the levels of gene expression. Based on m^6^A modification sites, RNA transcripts were classified into subgroups. As shown in (Figure 6D), m^6^A modifications of RNA transcripts in CDS, 5′UTR or 3′UTR do not differ with gene expression. Through studying m^6^A-modified sites and relative expression levels of genes, revealing that the genes have three or four modified sites appears to be more abundant in contrast with other m^6^A-modified sites (Figure 6E). Furthermore, we implemented qRT-PCR to confirm the expression of differentially methylated genes between adipocyte and preadipocyte. The mRNA expression pattern was consistent with the RNA-seq data (Supplementary Figure S4A–B), which confirms the validity of our transcriptome results.

**TABLE 1:** List of 28 genes with significant changes in m^6^A and mRNA transcript abundance in yak adipocyte as compared with preadipocytes.

Gene name	Pattern	m^6^A level change				mRNA level change	
		Peak region	Peak start	Peak end	diff.p	log2(fc)	*p*-value
QPRT	Hyper-up	Exon	491048	491299	0.03	2.30	0.00
BCL2L11	Hyper-up	5′ UTR	1175002	1175378	0.03	2.15	0.04
PER1	Hyper-up	3′ UTR	235431	236437	0.02	2.22	0.00
KLHL29	Hyper-up	3′ UTR	4950507	4950957	0.02	1.44	0.02
KLF9	Hyper-up	5′ UTR	1298633	1299052	0.01	2.18	0.00
ZNF395	Hyper-up	3′ UTR	730872	731331	0.01	2.25	0.00
ZNF608	Hyper-up	Exon	980374	980553	0.04	2.38	0.00
MTERF4	Hyper-up	3′ UTR	726115	726234	0.01	1.32	0.02
CD247	Hypo-down	3′ UTR	33979	34575	0.01	−1.78	0.02
SLCO5A1	Hypo-down	Exon	1066324	1066623	0.01	−2.00	0.01
AFAP1L2	Hypo-down	Exon	854,733	856,548	0.04	−3.48	0.02
CENPF	Hypo-down	Exon	1363460	1363640	0.01	−3.67	0.00
USP43	Hypo-down	3′ UTR	129792	130001	0.01	−3.08	0.00
ARHGEF28	Hypo-down	Exon	175098	178009	0.01	−2.81	0.00
ARAP3	Hypo-down	Exon	442290	442350	0.03	−3.53	0.00
PHF19	Hyper-down	3′ UTR	273548	273938	0.00	−1.29	0.04
ADAMTSL1	Hyper-down	3′ UTR	142570	142929	0.00	−1.99	0.01
PLD3	Hyper-down	3′ UTR	118422	118482	0.01	−1.16	0.05
CDCA8	Hyper-down	3′ UTR	4260517	4260782	0.01	−2.38	0.04
PLEKHA6	Hyper-down	Exon	333151	338353	0.01	−1.76	0.02
SHANK1	Hyper-down	Exon	403394	404020	0.01	−2.14	0.00
SHANK1	Hyper-down	Exon	406035	406274	0.01	−2.14	0.00
CENPF	Hyper-down	Exon	1341226	1357574	0.01	−3.67	0.00
B4GALNT1	Hyper-down	3′ UTR	320399	320607	0.01	−2.38	0.04
TEAD4	Hyper-down	3′ UTR	240855	242598	0.01	−1.50	0.03
RHBDF2	Hyper-down	3′ UTR	274815	275085	0.03	−1.86	0.02
UHRF1	Hyper-down	Exon	560816	563952	0.02	−2.62	0.01
FOXO1	Hypo-up	3′ UTR	475509	475778	0.01	1.21	0.05
LOC102267107	Hypo-up	3′ UTR	311297	311596	0.01	1.49	0.02

### Differentially Methylation Modification is Linked to the Translation of Genes

**FIGURE 7:**
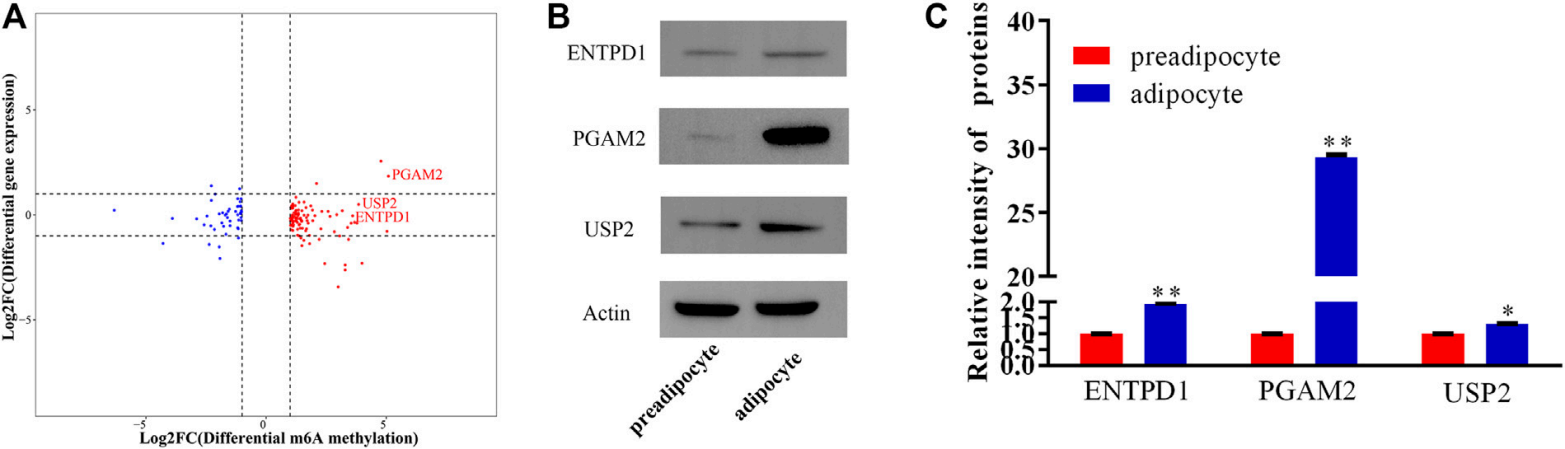
The analysis of genes with significant differences in methylation and non-significant differences expression during yak adipocytes differentiation. **(A)** Four quadrant plot showing the genes with significant differences in methylation and non-significant differences expression. **(B,C)** The proteins of USP2, ENTPD1, and PGAM2 were expressed in preadipocytes and adipocytes (SEM, * *p* < 0.05;** *p* < 0.01).

Previous research indicates that RNA methylation plays an essential role in the translation of mRNA. Therefore, to reveal the influence of RNA methylation on mRNA translation, we explored the metagene with significant differences for methylation and nonsignificant differences in gene expression during yak preadipocyte differentiation. There were 155 genes with significant differences in methylation, and nonsignificant differences in expression existed in preadipocytes and adipocytes (Figure 7A; Supplementary File S7). To predict the function of these genes, GO and KEGG analyses were performed. These genes are mainly allocated to organism development, DNA binding, canonical Wnt signaling pathway, citrate cycle (TCA cycle), and calcium signaling pathway (Supplementary Figure S5A–B; Supplementary Files S8, S9). Therefore, the candidate genes were selected from the top 10 genes (Table 2) with the peck fold change for Western blot. Interestingly, the protein expression levels (ENTPD1, USP2, and PGAM2) were substantially higher in the adipocyte than the preadipocyte group (Figure 7B,C). The findings indicate that RNA methylation not only may regulate mRNA expression, but also effect mRNA translation during yak preadipocyte differentiation.

**TABLE 2:** List of 10 genes with significant difference in m^6^A and nonsignificant difference expression in yak adipocyte as compared with preadipocyte.

Gene name	m^6^A level						mRNA level	
	Peak region	Peak start	Peak end	diff.log2.fc		diff.p	log2(fc)	*p*-value
PGAM2	Exon	437745	438043		5.09	0.04	1.85	1
LOC102285565	5′UTR	38451	38681		5.03	0.00	−0.78	0.44
LOC102280847	5′UTR	101406	101799		4.78	0.00	2.57	0.28
NRARP	Exon	501948	502122		3.99	0.00	−2.30	1
USP2	Exon	2422394	2422633		3.85	0.00	0.50	0.62
LOC102284166	5′UTR	252439	252618		3.78	0.02	−0.38	0.57
GPR135	Exon	30403	30553		3.68	0.02	−0.35	1
ENTPD1	5′UTR	638635	638694		3.60	0.01	−0.04	1.00
PRR22	Exon	80,040	80129		3.54	0.03	−0.39	0.76
ZFPM1	Exon	1465574	1465753		3.43	0.00	−1.16	0.24

## Discussion

The harsh environment of the Qinghai-Tibet Plateau encourages the yak to develop a special mechanism for energy metabolism. As an organ for energy metabolism, adipose tissue plays a crucial role in this process. To date, it is found that epigenetic regulation is engaged in various biological processes, including embryo development, stem cell self-renewal, DNA damage response, primary miRNA processing, and energy metabolism (Wu and Sun, 2006; Shi and Wu, 2009; Donohoe and Bultman, 2012; Li et al., 2013; Wang et al., 2013). In recent years, as the most extensive and plentiful internal modification on mRNAs, m^6^A modification is a major focus in the area of epigenetic regulation (Niu et al., 2013). Furthermore, the potential roles of m^6^A modification in most domestic animals, and especially for adipogenic differentiation, remained largely unknown. For the first time, our study establishes a comprehensive transcriptome-wide pattern of m^6^A modification in yak preadipocyte and adipocyte using MeRIP-Seq technology to explore the function of m^6^A modification in bovine adipogenic differentiation. Our findings show that yak mRNA m^6^A sites were primarily located in CDS, 5′UTRs and 3′UTRs, and the distribution semblable with humans and mice (Dominissini et al., 2012; Meyer et al., 2012), suggesting that, in mammalian transcriptomes, the overall distribution of m^6^A sites is similar. Besides this, Luo et al. reveal that m^6^A modifications were also enriched near the start codons of Arabidopsis (Luo et al., 2014). Thus, the distribution of m^6^A modification has various forms in different species. The m^6^A located at mRNA 5′UTR and 3′UTR of yak differ from mice and chickens (Luo et al., 2019; Cheng et al., 2021). We found m^6^A more enrichment in 3′UTR compared with 5′UTR, which contrasts with other mammals (Luo et al., 2019; Wang et al., 2019). The high-level of m^6^A methylation located in 3′UTR may be associated with mRNA stability, selective polyadenylation, signaling transport, and translocation (Shen et al., 2016; Yue et al., 2018). In addition, the m^6^A modification on the 3′UTR plays a regulatory element role for protein translation by recruiting specific factors to these m^6^A sites for RNA transport or protein synthesis (Niu et al., 2013; Wang et al., 2014). This may be one of the reasons causing a potential positive correlation between the degree of m^6^A methylation and transcript levels. Otherwise, the current study finds an m^6^A peak relatively increased at mRNA 5′UTR in Ad compared with Pread 0. The m^6^A located at mRNA 5′UTR can improve its cap-independent translation under heat shock (Meyer et al., 2015; Zhou et al., 2015). This indicates that the higher m^6^A signal at 5′UTR may promote mRNA translation during yak preadipocyte differentiation. Further, in our study, approximately 80% of the methylated transcripts included one or two m^6^A peaks, and about 20% of the methylated transcripts included three or more than three m^6^A peaks. The ratio is higher than in humans (5.5%) (Dominissini et al., 2012), pigs (10%) (Wang et al., 2018), chickens (5%) (Cheng et al., 2021), and mice (10%) (Luo et al., 2019). This phenomenon may be due to the more rapid rate of lipid metabolism in yaks, which is consistent with a previous study that cells and tissues with greater proliferation and differentiation capacity may require higher levels of m^6^A methylation to adapt to faster growth and development (Tao et al., 2017). According to previous studies, the consistent motif pattern of “RRACH” was over-represented in the m^6^A motif sequence area (Harper et al., 1990; Dominissini et al., 2012; Meyer et al., 2012). Accordingly, in comparison with previous studies (Dominissini et al., 2012; Meyer et al., 2012), the consensus motif GGACU sequence in the yak transcriptome was appropriately identified, revealing that RNA adenosine methylation was conserved in mammals.

Earlier studies indicate that m^6^A modification is closely related to gene expression (Meyer et al., 2012; Fu et al., 2014; Yue et al., 2015; Chen et al., 2020). Jean-Michel Fustin et al. report that *METTL3* depletion inhibited the export mRNA (Jean-Michel et al., 2013), and Guanqun Zheng et al. report that depletion of *ALKBH5* increased the export of mRNA to the cytoplasm (Zheng et al., 2013), suggesting m^6^A promotes the export of mRNA and modulates gene expression (Zhao et al., 2017). In HeLa cells, YTHDC1 was discovered to interact with SRSF3 and nuclear RNA export factor 1 (NXF1) to promote the export of m^6^A-modified mRNA out of the nucleus (Roundtree IA. et al., 2017). These results indicate a potential positive association between the degree of m^6^A methylation and the transcript level. In the present study, the genes *METTL3*, *WTAP*, *METTL14*, *FTO*, *ALKBH5*, and *YTHDC1/2* were dramatically upregulated in adipocytes than the preadipocytes, and the majority of modified m^6^A genes were expressed at a medium level with a positive relationship in gene expression and m^6^A methylated modification. Our findings are in agreement with Chen et al., who reveal that m^6^A modifications tend to have a positive correlation with mRNA expression in clear cell renal cell carcinoma (Chen et al., 2020). These findings show that m^6^A methylation affects gene expression by controlling post-transcription regulation. The m^6^A-reader protein-containing YTH structural domain 2 (YTHDC2) can preferentially bind m^6^A within the consensus motif and improve the translation efficiency of mRNA (Yang et al., 2018). Interestingly, *YTHDC2* was significantly upregulated during yak preadipocyte differentiation. Therefore, we speculate that m^6^A methylation modification not only influences mRNA expression but also may regulate mRNA translation during yak preadipocyte differentiation. Consequently, the genes with significant differences in methylation and nonsignificant differences in expression were detected in this study. Intriguingly, the results of Western blot revealed that the expression of ectonucleotidases CD39 (ENTPD1), ubiquitin-specific protease-2 (USP2), and phosphoglycerate mutase 2 (PGAM2) were significantly elevated in adipocytes compared with preadipocytes. Previous studies report that USP2 can influence the stabilization of fatty acid synthase (FAS), and 3,3′-diindolylmethane inhibits adipogenesis in preadipocytes by targeting USP2 activity (Graner et al., 2004; Yang et al., 2017). Enjyoji et al. reveal that entpd1-deficient mice have impaired glucose tolerance, reduced insulin sensitivity, and significantly elevated plasma insulin levels (Enjyoji et al., 2008). PGAM2 plays an important role in glycolysis, muscle growth and development, and organism physiological balance (Qiu et al., 2008; Mikawa et al., 2021). Accordingly, it is logical to conclude that m^6^A methylation modification exerts an essential role through affecting the translation of mRNA during yak preadipocyte differentiation. Nevertheless, further study is needed to verify the conjecture.

GO analysis explored the differentially methylated genes, which participated in the transcript regulation with a variety of transcription factors by RNA polymerase II. For example, FOXO1 identified as a Forkhead transcription factor controlling the differentiation of adipocytes (Nakae et al., 2003) and many members of the ZNF family considered as the crucial eukaryotic transcription factors involved in adipogenic metabolism (Wei et al., 2013), indicating m^6^A methylation participates in lipid metabolism. The KEGG pathway analysis revealed that the signaling pathway of differentially methylated genes is closely related to adipose metabolisms, such as the FoxO signaling pathway, Ether lipid metabolism, Glycerophospholipid metabolism, and Hippo signaling pathway-multiple species. In particular, *FOXO1* was further found to be involved in the FoxO signaling pathway, which demonstrated the importance of adipocyte differentiation (Nakae et al., 2003). As a TEA domain family transcription factor, *TEAD4* was selected from Hippo signaling pathway-multiple species, which recruits the cofactors VGLL4 and CtBP2 to inhibit murine adipogenesis (Zhang W. et al., 2018). To summarize the above findings, we concluded that activating the FoxO and Hippo signaling pathways through m^6^A methylated gene may perform a key function during the differentiation of yak adipocytes.

Integrated analysis of m^6^A-seq and mRNA-seq data exposed that 28 significant change genes exist in the adipocyte group with differently methylated m^6^A sites compared with preadipocyte. Several of the genes are confirmed to regulate adipose metabolism and adipogenic differentiation, such as *ZNF395*, *KLF9*, *TEAD4*, *FOXO1*, and *UHRF1*. *ZNF395*, the mRNA of which is hypermethylated and the expression upregulated in the adipocyte group compared with the preadipocyte group. As a member of the C2H-type Zinc finger proteins, *ZNF395* is classified as Papillomavirus-binding factor and Huntington disease gene regulatory region binding protein 2 (Tanaka et al., 2004). Experiments of loss and gain function demonstrate that *ZNF395* interacts with *PPARG2* to modulate the transcriptional regulatory pathway that may be necessary for preadipocyte differentiation (Hasegawa et al., 2013). Besides this, previous literature reports that mesenchymal stem cells were cotransduced with *ZNF395* and *PPARG2* enhanced the endogenous expression of *PPARG2* and *C/EBPα*, which are necessary for adipocyte differentiation (Sichtig et al., 2007; Hasegawa et al., 2013). In addition to that, it is reported that Krüppel-like factor 9 (*KLF9*), deemed to be the basic transcription element-binding protein-1 (BTEB1), could transactivate *PPARγ2* to regulate adipogenesis in the 3T3-L1 cell line (Pei et al., 2011). Besides this, Kimura Hiroko et al. find that *KLF9* triggered the early stage of adipogenesis by promoting the *C/EBPβ* gene expression in 3T3-L1 cells (Kimura and Fujimori, 2014). Ubiquitin-like with PHD and RING finger domains 1 (*UHRF1*) is widely documented to promote cell proliferation. Additionally, a study revealed that *UHRF1* facilitates the proliferation of human adipose-derived stem cells and represses adipogenesis via inhibiting peroxisome proliferator-activated receptor γ (Chen et al., 2019). These findings suggest that m^6^A modifications may perform an essential role during yak adipocyte differentiation.

## Conclusion

Current findings display that the m^6^A profiles and distribution patterns in the yak transcriptome. Besides this, functional enrichment analysis of differentially methylated genes reveal that several candidate genes participated in lipid metabolic pathways, suggesting that m^6^A methylation modifications are involved in the modulation of yak preadipocyte differentiation. Furthermore, we also explore the correlation between m^6^A methylation and the level of gene expression or mRNA translation, indicating a potential regulatory mechanism for m^6^A in adipocyte differentiation. These results provide additional knowledge of m^6^A methylation in adipose tissues, and it set the foundation for further understanding its possible roles and regulatory mechanisms, which could be helpful for exploration the yak adaptive mechanism in the harsh environment.

## Data Availability

The data was submitted to the data base of the Sequence Read Archive (SRA). The appropriate number for accession is PRJNA649748.
