# Supplementary figures and images for: Integrated Study of Transcriptome-wide m6A Methylome Reveals Novel Insights Into the Character and Function of m6A Methylation During Yak Adipocyte Differentiation
Source: Front Cell Dev Biol. 2021 Dec 3;9:689067. doi: 10.3389/fcell.2021.689067 (PMC8678508; doi:10.3389/fcell.2021.689067)

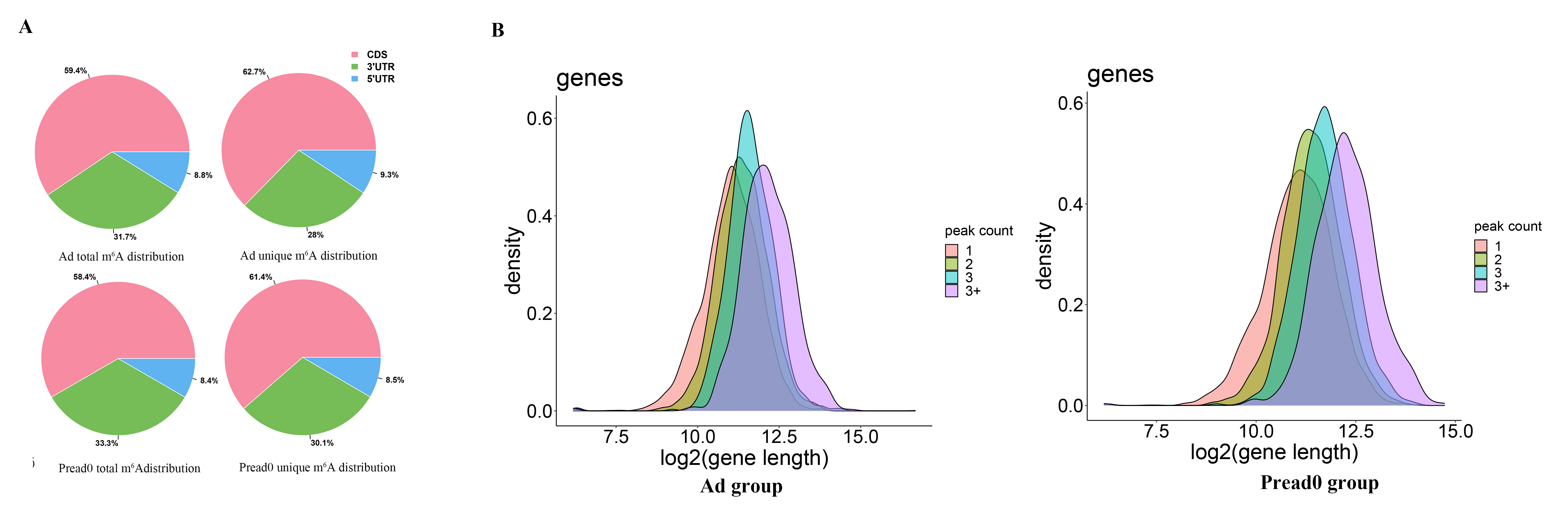

Supplement: Supplementary file 4 [file Image3.TIF]

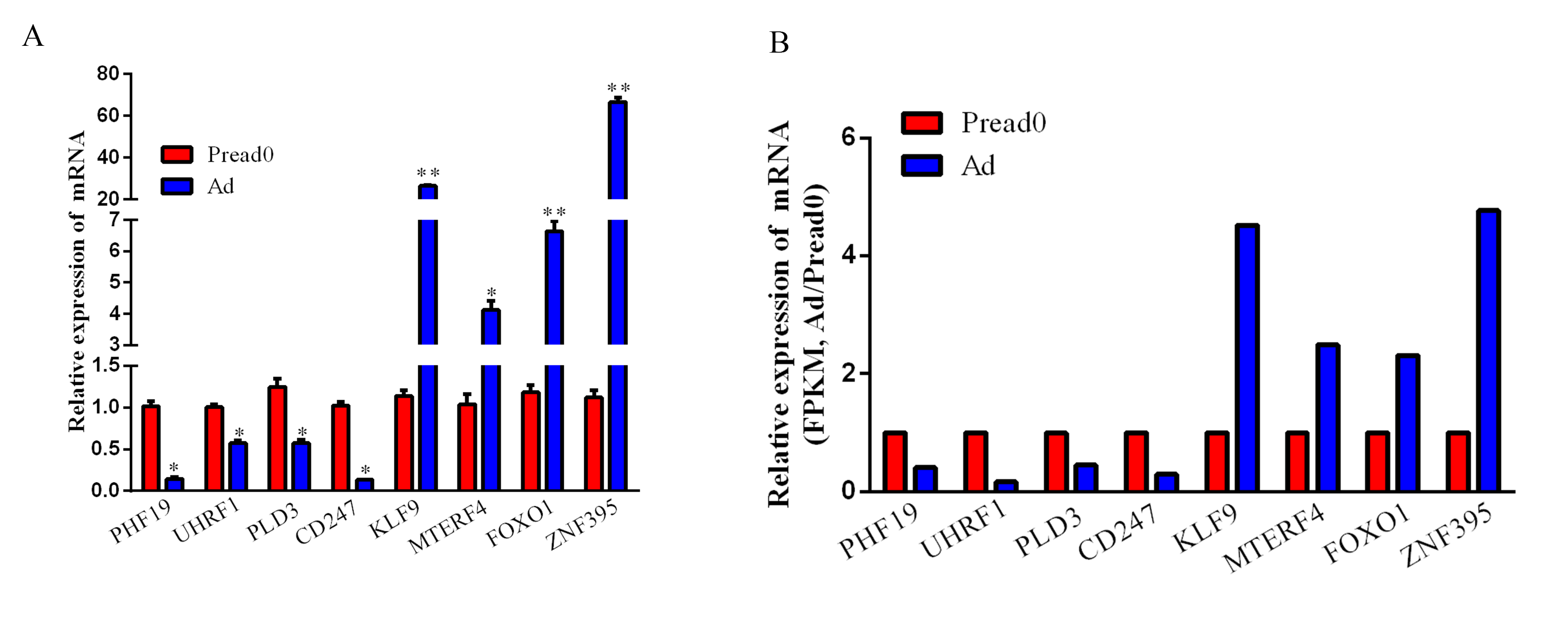

Supplement: Supplementary file 5 [file Image4.TIF]

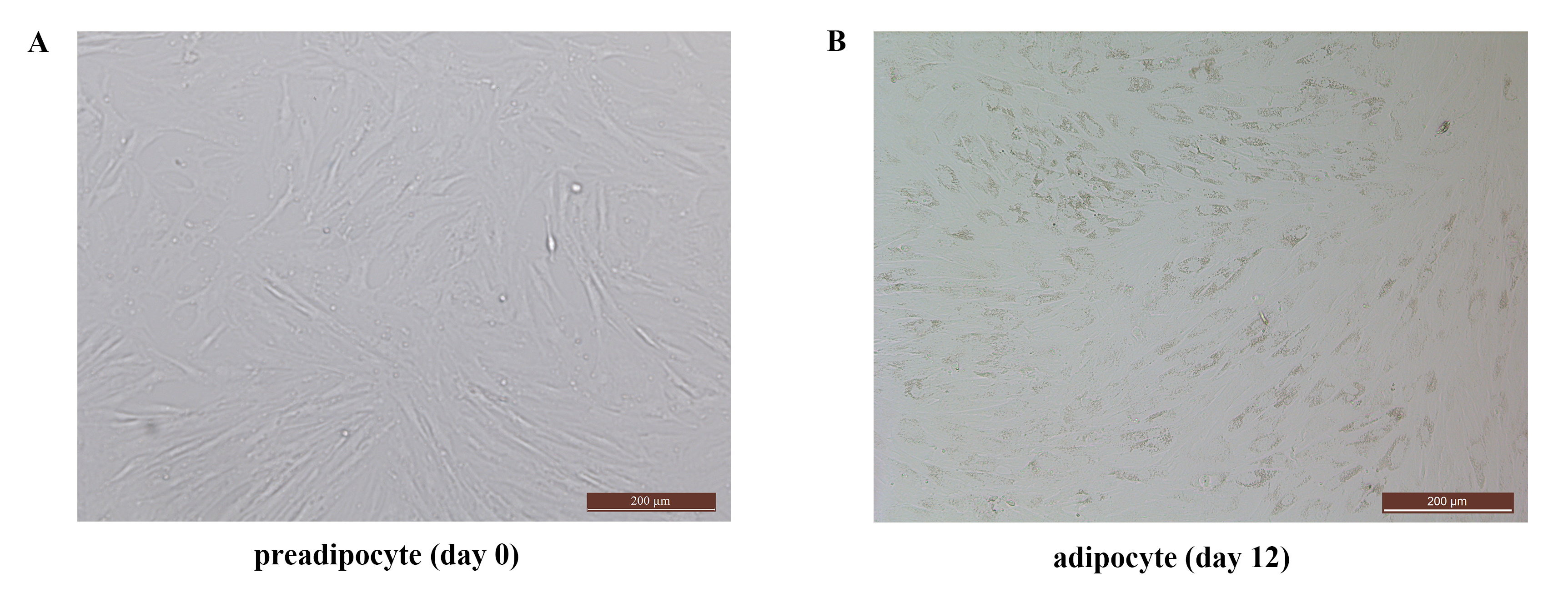

Supplement: Supplementary file 8 [file Image1.TIF]

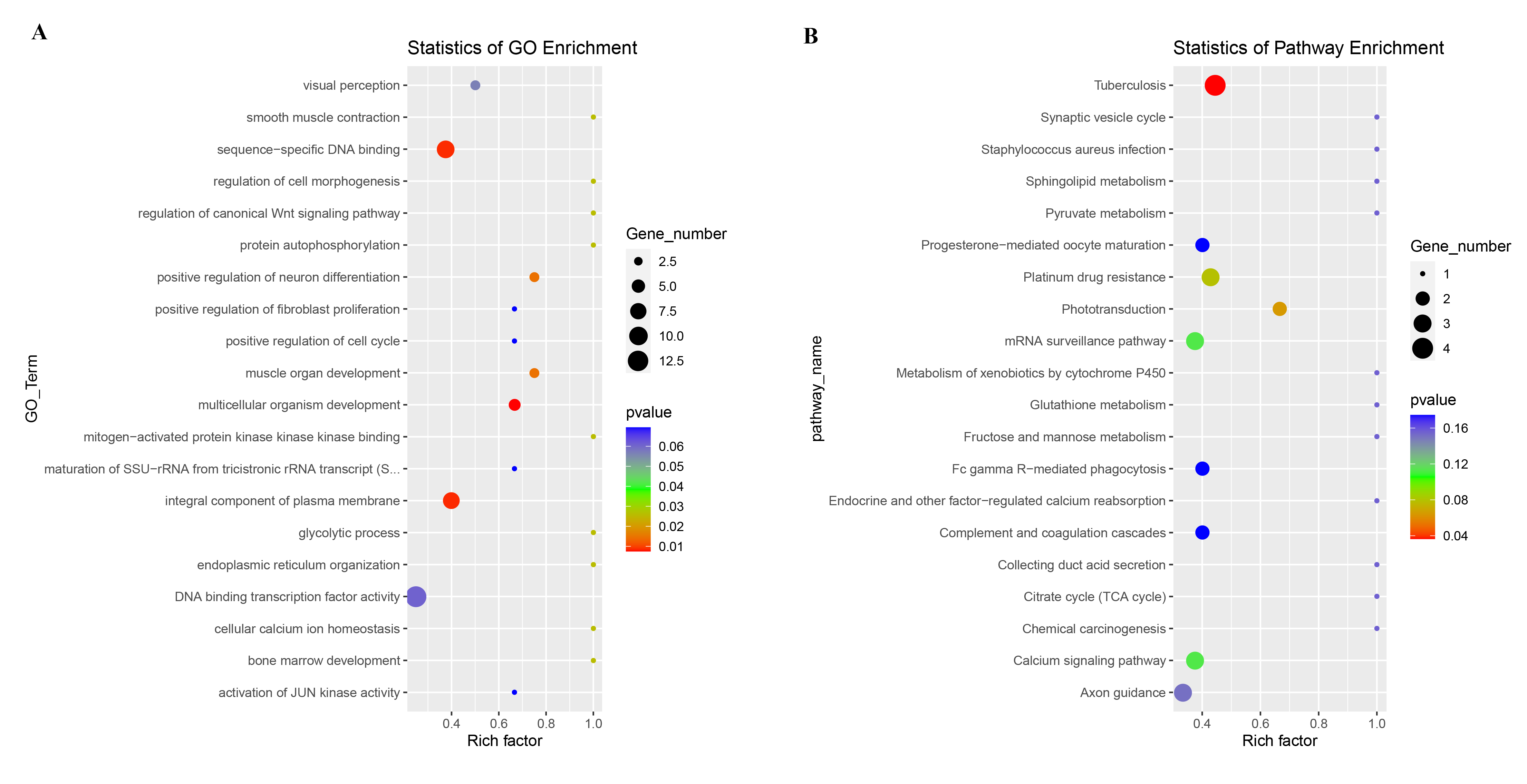

Supplement: Supplementary file 13 [file Image5.TIF]
