# Supplementary material for: Integrated Study of Transcriptome-wide m6A Methylome Reveals Novel Insights Into the Character and Function of m6A Methylation During Yak Adipocyte Differentiation
Source: Front Cell Dev Biol. 2021 Dec 3;9:689067. doi: 10.3389/fcell.2021.689067 (PMC8678508; doi:10.3389/fcell.2021.689067)
Supplement: Supplementary file 6 [file Table12.DOCX]

**TABLE S2∣**Summary of sequencing reads.

| **Sample_ID** | **Raw_Reads** | **Raw_Bases** | **Valid_Reads** | **Valid_Bases** | **Valid%** | **Q30%** | **GC%** | **Mapped reads%** |
| --- | --- | --- | --- | --- | --- | --- | --- | --- |
| Pread0_1_IP | 55184142 | 8.28G | 47883370 | 5.86G | 70.78 | 95.02 | 55.28 | 87.96 |
| Pread0_2_IP | 55938860 | 8.39G | 50587068 | 6.14G | 73.21 | 95.2 | 54.55 | 90.00 |
| Pread0_3_IP | 69039438 | 10.36G | 68104878 | 9.54G | 92.12 | 94.76 | 51.83 | 95.97 |
| Ad_1_IP | 57479666 | 8.62G | 50401630 | 5.76G | 66.75 | 94.91 | 55.03 | 89.23 |
| Ad_2_IP | 63541270 | 9.53G | 55415226 | 6.39G | 67.05 | 95.05 | 55.37 | 90.09 |
| Ad_3_IP | 67513122 | 10.13G | 66631086 | 9.31G | 91.98 | 94.63 | 51.85 | 96.12 |
| Pread0_1_input | 60201678 | 9.03G | 55029224 | 6.27G | 69.4 | 95.55 | 56.28 | 88.26 |
| Pread0_2_input | 60834330 | 9.13G | 56966702 | 6.15G | 67.45 | 95.63 | 56.13 | 90.61 |
| Pread0_3_input | 63988228 | 9.60G | 63228712 | 8.87G | 92.39 | 94.58 | 49.23 | 96.57 |
| Ad_1_input | 67078194 | 10.06G | 59766498 | 6.26G | 62.18 | 95.55 | 54.38 | 91.01 |
| Ad_2_input | 61451380 | 9.22G | 55766110 | 6.00G | 65.1 | 95.65 | 55.35 | 90.38 |
| Ad_3_input | 66057648 | 9.91G | 65049044 | 9.11G | 91.93 | 94.47 | 49.01 | 96.57 |
