# Supplementary material for: Integrated Study of Transcriptome-wide m6A Methylome Reveals Novel Insights Into the Character and Function of m6A Methylation During Yak Adipocyte Differentiation
Source: Front Cell Dev Biol. 2021 Dec 3;9:689067. doi: 10.3389/fcell.2021.689067 (PMC8678508; doi:10.3389/fcell.2021.689067)
Supplement: Supplementary file 12 [file Table11.DOCX]

**TABLE S1∣**Sequence specific primers were used in this study. F: forward, R: reverse. β-actin was used as endogenous control genes for mRNA.

| **Gene** | **Primer sequences (5' to 3')** | **TM/ oC** |
| --- | --- | --- |
| METTL3 | F: TAGCCAAGGAGCCAACCAAG  R: TCTTGAACTTGAGCCCGACC | 59 |
| METTL14 | F: TCTGGGGAAGGATTGGACCT  R: CCCGTCTGTGCTACGCTTTA | 59 |
| WTAP | F: GTCTGGATTTCACAGGGAGGG  R: CAGGTTTCTCCTCTTGCGGG | 59 |
| FTO | F: GCACAAGCACGGCTGTTTAT  R: TTGACAGGCGGCAGCTATTT | 59 |
| ALKBH5 | F: ACGAGATTAGATGCACCCCG  R: CTGTTGTTTCCCGACAGACG | 61 |
| YTHDC1 | F: GCATTCTCAGCCCCGATCAA  R: GTGGTGGTGGTCCCATGTTA | 64.5 |
| YTHDC2 | F: GCCAGAGGCAGCCAGTTTAT  R: CAGATGGCTGCTGTAAGCCT | 54 |
| PLD3 | F: AGGAACCTGCCAGCGAACT  R: CCAGAAGGACCCAACGAG | 61.4 |
| CD247 | F: TCTTCATCTACGGCGTTATTG  R: GGGTTCTTCTTCCTCGGTTT | 56 |
| PHF19 | F: GGTGGTCCTGGAGAATGG  R: GCACGCCCAGGTTGTAG | 63.3 |
| UHRF1 | F: CCGCAAGCACAGCAAGTATG  R: GTCAGCCCCAGCTTCTTGAT | 61.4 |
| ZNF395 | F: GAGACTGACCCAGACGCTTT  R: GAAATCCTCCTCCCGCTTGA | 61.4 |
| MTERF4 | F: GGTGCTTTACTGTTGCCCTG  R: GCAGGTCCGTCTTCACAATG | 61.4 |
| FOXO1 | F: GCTCCTGGTGGATGCTCAAT  R: AGAGCCTCCCGCTAATGGTA | 61.4 |
| KLF9 | F: TCCTCCCATCTCAAAGCCCA  R: GTCGGAGCGGGAGAACTTTT | 61.4 |
| PPARγ | F:CATTTCCACTCCGCACTA  R:GGGATACAGGCTCCACTT | 60.3 |
| FABP4 | F:CATTAAATCCGAAAGCA  R:CTCATAAACTCTGGTGGC | 60.3 |
| C/EBPα | F:GCGGCAACGACTTTGACTACCC  R:CTGCTTCGCTTCGTCCTCCTC | 60.3 |
| β-actin | F:GCAGGTCATCACCATCGG  R:CCGTGTTGGCGTAGAGGT | 60.3 |
