# Supplementary material for: Integrated Study of Transcriptome-wide m6A Methylome Reveals Novel Insights Into the Character and Function of m6A Methylation During Yak Adipocyte Differentiation
Source: Front Cell Dev Biol. 2021 Dec 3;9:689067. doi: 10.3389/fcell.2021.689067 (PMC8678508; doi:10.3389/fcell.2021.689067)
Supplement: Supplementary file 17 [file Table10.DOCX]

**Supplementary Material**

**Integrated study of transcriptome-wide m^6^A methylome reveals novel insights into the character and function of m^6^A methylation during yak adipocytes differentiation**

Yongfeng Zhang ^1,2^, Chunnian Liang ^1^, Xiaoyun Wu ^1^, Jie Pei ^1^ , Xian Guo ^1^, Min Chu ^1^, Xuezhi Ding ^1^, Pengjia Bao^1^, Qudratullah Kalwar^1^, Ping Yan ^1,2*^

^1^Key Laboratory of Yak Breeding Engineering Gansu Province, Lanzhou Institute of Husbandry and Pharmaceutical Sciences, Chinese Academy of Agricultural Sciences, Lanzhou 730050, Gansu, China

^2^State Key Laboratory of Grassland Agro-Ecosystems, College of Pastoral Agriculture Science and Technology, Lanzhou University, Lanzhou 730000, Gansu, China

*** Correspondence:**

Corresponding author: Ping Yan (email: [pingyanlz@163.com](mailto:pingyanlz@163.com); TEL: 0931-2164180)

**FIGURE S1∣**Yak preadipocytes were induced differentiation at (A) day 0 and (B) day 12.

**FIGURE S2∣**The RNA species of transcripts. Red circles represent mRNA, orange circles represent misc_RNA, blue circles represent ncRNA, green circles represent pseudogene, and purple circles represent tRNA, respectively.

**FIGURE S3∣**The Distribution of m^6^A Modification in Yak Transcriptome. (A) Venn diagram showing the m^6^A peaks for transcripts of two groups. (B) Density plots showing that the longer of gene length has more m^6^A peaks.

**FIGURE S4∣**qPCR tests of eight differentially expressed genes modified by m^6^A in preadipocyte and adipocyte. (A) The relative mRNA levels were determined by qPCR of eight genes in both groups and (B) the genes change levels based on RNA-seq data.

**FIGURE S5∣**The enrichment analysis for genes of significant differences in methylation and non-significant differences in preadipocyte and adipocyte. (A) The top 20 GO terms of genes with significant differences in methylation and non-significant differences between preadipocyte and adipocyte. (B) The top 20 enriched pathways for the genes of significant differences in methylation and non-significant differences between preadipocyte and adipocyte.

**Supplementary File 1.** Go analysis of differentially methylated m^6^A peaks represented genes.

**Supplementary File 2.** KEGG analysis of differentially methylated m^6^A peaks represented genes.

**Supplementary File 3.** The genes (KLF9, FOXO1 and UHRF1) of m^6^A and mRNA transcript abundance in yak adipocyte as compared to preadipocyte.

**Supplementary File 4.** Clustered heat map for the top 100 most differentially expressed genes.

**Supplementary File 5.** The top 20 GO enrichment terms for differentially genes.

**Supplementary File 6.** The top 20 KEGG enrichment pathways for differentially genes.

**Supplementary File 7.** List of 155 genes with significant differences in methylation and non-significant differences expression in yak adipocyte as compared to preadipocyte.

**Supplementary File 8.** The GO enrichment terms for genes of significant differences in methylation and non-significant differences.

**Supplementary File 9.** The KEGG enrichment terms for genes of significant differences in methylation and non-significant differences.
